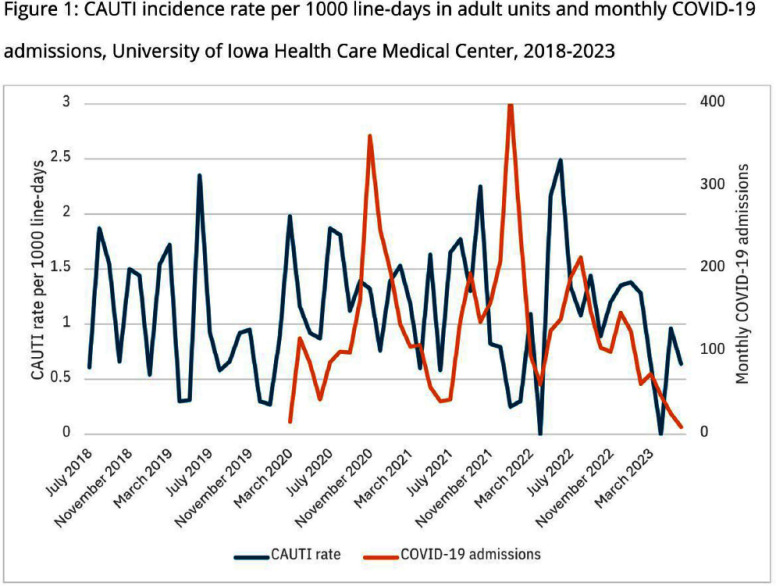# Descriptive Epidemiology of Catheter-Associated Urinary Tract Infections at University of Iowa Health Care Medical Center

**DOI:** 10.1017/ash.2025.283

**Published:** 2025-09-24

**Authors:** Lauren Kloft, Takaaki Kobayashi, Alexandra Trannel, Karen Brust, Alexandre Marra, Oluchi Abosi, Nicole Wiltfang, Beth Hanna, Jaime Murphy, Daniel Diekema

**Affiliations:** 1 State Hygienic Laboratory at the University of Iowa; 2 University of Kentucky; 3 University of Iowa Hospitals & Clinics; 4 University of Iowa Health Care; 5 University of Iowa Hospitals & Clinics/ Hospital Israelita Albert Einstein; Mariana Kim Hsieh, University of Iowa Health Care; 6 University of Iowa Hospital and Clinics; 7 University of Iowa Health Care; 8 The University of Iowa Hospitals and Clinics; 9 University of Iowa Hospitals and Clinics; 10 University of Iowa Carver College of Medicine

## Abstract

**Background:** Catheter-associated urinary tract infections (CAUTIs) are among the most common healthcare-associated infections (HAIs), often resulting in prolonged hospital stays, increased healthcare costs, and additional clinical interventions. The COVID-19 pandemic introduced new challenges to infection prevention, with global reports indicating increased rates of certain HAIs, such as ventilator-associated pneumonia and bloodstream infections, due to healthcare strain and the intensified use of invasive devices. However, trends in CAUTI rates during the pandemic varied across healthcare settings. **Methods:** This retrospective study was conducted at the University of Iowa Health Care Medical Center, an 866-bed academic hospital, from 2018 to 2023. Manual chart reviews of CAUTI cases reported to the National Healthcare Safety Network (NHSN) were performed to collect data on patient demographics, medical histories, catheter usage, and infection prevention practices. CAUTI incidence was analyzed over time and compared with monthly COVID-19 admission rates. **Results:** A total of 226 CAUTI cases were identified during the study period. The average CAUTI rate per 1,000 catheter line-days declined from 1.23 in 2019 to 0.85 in 2020, but increased to 1.28 in 2021, coinciding with COVID-19 surges (Figure 1). The median patient age was 61 years, with females accounting for 56% of cases. Foley catheters were already in place upon admission in 24% of cases. Non-intensive care unit (ICU) inpatient settings accounted for 24% of catheter placements, while ICUs accounted for 18%. Additionally, 16% of cases originated from the operating room, and 7% from the emergency department. Neurologic disease was the most common admission diagnosis (27%), followed by cardiovascular disease (13%) and Hematologic/Oncologic disease (13%). Twenty six percent of cases were incontinent of urine and 24% of stool. Comorbidities included immunocompromised status (20%) and diabetes (36%). The primary indication for Foley catheter use was monitoring intake and output (42%). Of the 226 cases, 61% of patients were clinically considered to have a UTI. In-hospital mortality was 22%. **Conclusion:** The findings from this study provide insights into factors contributing to CAUTI at our institution. Fluctuations in CAUTI incidence, particularly during the COVID-19 pandemic, underscore the need for robust infection prevention strategies. The finding that only 61% of cases required treatment suggests urine cultures were often obtained inappropriately or positive results were not used in selected situations. This highlights an opportunity for diagnostic stewardship to improve urine culture practices. Addressing identified risk factors and enhancing catheter management are critical to reducing CAUTI incidence and improving patient outcomes.

**Figure:**